# The apTTTG distance decreases linearly with knee flexion: A dynamic CT‐based analysis in patients with patellofemoral instability

**DOI:** 10.1002/jeo2.70893

**Published:** 2026-09-12

**Authors:** Sebastian Schmidt, Alexander Bumberger, Miho J. Tanaka

**Affiliations:** ^1^ Department of Orthopedic Surgery Brigham and Women's Hospital, Harvard Medical School Boston Massachusetts USA; ^2^ Department of Orthopaedic and Trauma Surgery, University Medical Centre Mannheim, Medical Faculty Mannheim University of Heidelberg Mannheim Germany; ^3^ Department of Orthopedics and Trauma Surgery Medical University of Vienna Vienna Austria; ^4^ Department of Orthopedic Surgery Mass General Brigham, Harvard Medical School Boston Massachusetts USA

**Keywords:** dynamic CT, patellofemoral alignment, patellofemoral instability, sagittal TT–TG

## Abstract

**Purpose:**

The anterior–posterior tibial tubercle–trochlear groove (apTTTG) distance is a radiographic parameter describing the sagittal alignment of the patellofemoral joint and has been proposed as an indirect estimate of patellofemoral contact pressure, but its dynamic behaviour across different degrees of knee flexion remains unclear. This study aimed to evaluate flexion‐dependent changes in apTTTG using dynamic kinematic computed tomography (DKCT) imaging and to compare its behaviour with other established patellofemoral alignment parameters.

**Methods:**

This study was designed as a retrospective analysis of prospectively enroled patients with unilateral patellofemoral instability who underwent bilateral DKCT imaging as part of their diagnostic evaluation. The apTTTG was measured as the antero‐posterior distance from the deepest point of the trochlear groove to the central prominence of the tibial tubercle. This was measured at 0°, 30° and 60° of flexion. Changes in apTTTG were compared across flexion states using nonparametric statistics. Linear, quadratic and generalized additive models (GAMs) were used to characterize the relationship between apTTTG and knee flexion. Multivariable models included morphologic risk factors for patellar instability, including tibiofemoral rotation angle (TFRA), TT–TG, patellar tilt, bisect offset and sulcus angle.

**Results:**

Forty‐two knees from 21 patients (mean age 22.5 ± 7.2 years) were included. The apTTTG decreased from −3.8 ± 5.7 mm in extension to −8.9 ± 7.4 mm at 30° and −17.8 ± 8.4 mm at 60° of flexion (*p* < 0.001). Linear regression demonstrated a strong inverse association between knee flexion and apTTTG, corresponding to a reduction of 0.29 mm per degree of knee flexion (*β* = −0.29, *R* = 0.75, *p* < 0.001). In contrast, rotational alignment and patellar positioning parameters exhibited their most pronounced changes between extension and midflexion. No other anatomical parameter independently predicted apTTTG.

**Conclusion:**

The apTTTG distance decreases linearly with increasing knee flexion, whereas other patellofemoral alignment parameters change predominantly between extension and midflexion.

**Level of Evidence:**

Level IV.

**Abbreviations**
ACL: anterior cruciate ligament
AIC: Akaike Information Criterion
apTTTG: anterior–posterior tibial tubercle–trochlear groove distance
BMI: body mass index
BO: bisect offset
CI: confidence interval
CT: computed tomography
DFO: distal femoral osteotomy
DKCT: dynamic kinematic computed tomography
edf: effective degrees of freedom
GAM: generalized additive model
HTO: high tibial osteotomy
ICC: intraclass correlation coefficient
IRB: institutional review board
MOWHTO: medial open‐wedge high tibial osteotomy
MRI: magnetic resonance imaging
PCL: posterior cruciate ligament
PFI: patellofemoral instability
SD: standard deviation
sTTTG: sagittal tibial tubercle–trochlear groove distance
TFRA: tibiofemoral rotation angle
TTO: tibial tubercle osteotomy
TT–PCL: tibial tubercle–posterior cruciate ligament distance
TT–TG: tibial tubercle–trochlear groove distance
VAS: visual analogue scale

## INTRODUCTION

Patellofemoral instability (PFI) is a multifactorial condition associated with patellar maltracking or dislocation, recurrent symptoms, functional impairment and potential long‐term degenerative changes, making accurate radiographic assessment of anatomical risk factors essential for diagnosis and treatment [5, 7, 9, 13, 17, 22].

In the past, radiographic evaluation of PFI has relied heavily on axial imaging measurements to evaluate the patellofemoral articulation. Accurate assessment of axial femorotibial alignment, patellar position and trochlear morphology is essential because these parameters influence patellar tracking, patellofemoral joint loading and the risk of recurrent instability; however, they primarily reflect static anatomical relationships assessed in the axial and coronal planes. Accordingly, tibiofemoral rotation, patellar tilt, bisect offset (BO), tibial tubercle–trochlear groove (TT–TG) distance, sulcus angle and tibial tubercle–posterior cruciate ligament (TT–PCL) are routinely evaluated to contextualize patellofemoral alignment and inform risk stratification and surgical planning [4, 6, 12, 14, 15].

The anterior–posterior tibial tubercle–trochlear groove (apTTTG) distance, also referred to as the sagittal TT–TG (sTTTG), has emerged as a novel parameter to evaluate the sagittal relationship between the tibial tubercle and the trochlear groove [1, 2, 26, 28]. Abnormal values have been associated with conditions like trochlear dysplasia and cartilage degeneration [1, 12, 14]. This measurement has been proposed to guide surgical planning during tibial tuberosity osteotomy and may estimate patellofemoral contact pressures [26, 28]. Therefore, accurate and reproducible assessment of this measurement is critical. While some studies suggest that other measurements associated with patellar instability, such as TT–TG distance, change with varying degrees of knee flexion, the behaviour of the apTTTG across different flexion angles remains unclear [2, 12, 26, 31, 32]. This uncertainty is clinically relevant, as flexion‐dependent changes could influence measurement reliability, interstudy comparability and the interpretation of apTTTG in surgical planning.

Therefore, this study aimed to determine whether knee flexion angle influences apTTTG distance across extension, midflexion and flexion, and whether this relationship is independent of established patellofemoral alignment parameters. It was hypothesized that apTTTG decreases with increasing knee flexion and that knee flexion angle is the primary determinant of apTTTG.

## METHODS

### Patient selection

This study represents a retrospective analysis of prospectively enroled patients evaluated for recurrent patellar instability between 2019 and 2020. Institutional review board approval was obtained prior to patient enrolment. All patients were assessed by the senior author (M. J. T.) and underwent standardized bilateral dynamic kinematic computed tomography (DKCT) imaging following failure of conservative treatment, with imaging performed for the purpose of presurgical planning. To minimize the influence of acute‐phase symptoms such as pain‐related muscle inhibition or restricted range of motion, all DKCT studies were acquired at least four weeks after the initial clinical presentation.

Inclusion criteria were patients with unilateral patellar instability who underwent bilateral four‐dimensional computed tomography (4DCT) imaging as part of their standard diagnostic evaluation. Contralateral knees were included in the repeated‐measures analysis but were not considered healthy controls, as they demonstrated radiographic features of trochlear dysplasia without a clinical history of patellar instability. Accordingly, the contralateral knees were interpreted as morphologically at‐risk but clinically stable knees within the same patients. Patients with failed soft tissue patellar stabilization surgery with symptomatic instability were also eligible for inclusion, as their persistent instability allowed evaluation of patellofemoral alignment under clinically relevant pathological conditions. Only patients with closed physes at the time of imaging were included. Medical records were reviewed to confirm the presence or absence of PFI for each knee.

Exclusion criteria included any prior bony procedures involving the distal femur or proximal tibia, such as tibial tubercle osteotomy (TTO), trochleoplasty, distal femoral osteotomy (DFO) or high tibial osteotomy (HTO); bilateral clinical PFI; acute primary stabilization due to osteochondral fractures; isolated patellofemoral pain without objective signs of instability; prior ligamentous knee surgery, including anterior cruciate ligament (ACL) or posterior cruciate ligament (PCL) reconstruction; clinical or magnetic resonance imaging (MRI)‐based evidence of ACL or PCL insufficiency, including a positive anterior or posterior drawer test; inflammatory joint disease; and radiographic evidence of advanced osteoarthritis defined as Kellgren–Lawrence grade >2. Patients were further excluded if DKCT imaging was incomplete, of insufficient quality or failed to capture full knee extension.

### Imaging evaluation

All patients underwent standardized DKCT imaging using a GE Revolution CT scanner in cine mode, which has been previously validated for the assessment of patellar tracking and patellofemoral alignment [27, 30]. During acquisition, patients were seated in the scanner gantry with their thighs gently stabilized to minimize rotational artifacts while allowing unrestricted active knee motion. No support roll was placed beneath the knee, and no anteriorly directed external force was applied to the tibia during image acquisition. Patients were instructed to perform continuous active knee flexion and extension for approximately 1.5 cycles over a 10‐s period, without any external resistance beyond gravity. Volumetric image data were captured at 0.5‐s intervals, yielding high‐resolution three‐dimensional datasets of the patellofemoral joint throughout the motion arc. Post‐processing was performed using multiplanar advanced visualization software (Visage 7, Version 7.1.15; Visage Imaging Inc.). Two independent observers (A. B. and S. S.), blinded to clinical status, conducted quantitative assessments of apTTTG, TT–TG distance and tibiofemoral rotation angle (TFRA) to characterize the relationship between the distal femur and tibia. BO and patellar tilt were measured to characterize patellar position, and sulcus angle was measured to characterize trochlear dysplasia. To reflect different stages of patellofemoral engagement, measurements were obtained at three key knee flexion phases: full extension (representing the disengaged phase), mid‐flexion around 30° (indicating initial bony engagement) and approximately 60° (reflecting full bony engagement). Because of the dynamic image acquisition protocol, the CT frame closest to each respective flexion angle was selected to ensure accurate alignment of measurement values with joint position. Except for the knee flexion angle, which was determined on sagittal reconstructions, all parameters were measured on axial CT images.

### Anterior–posterior TT–TG

**Figure 1:**
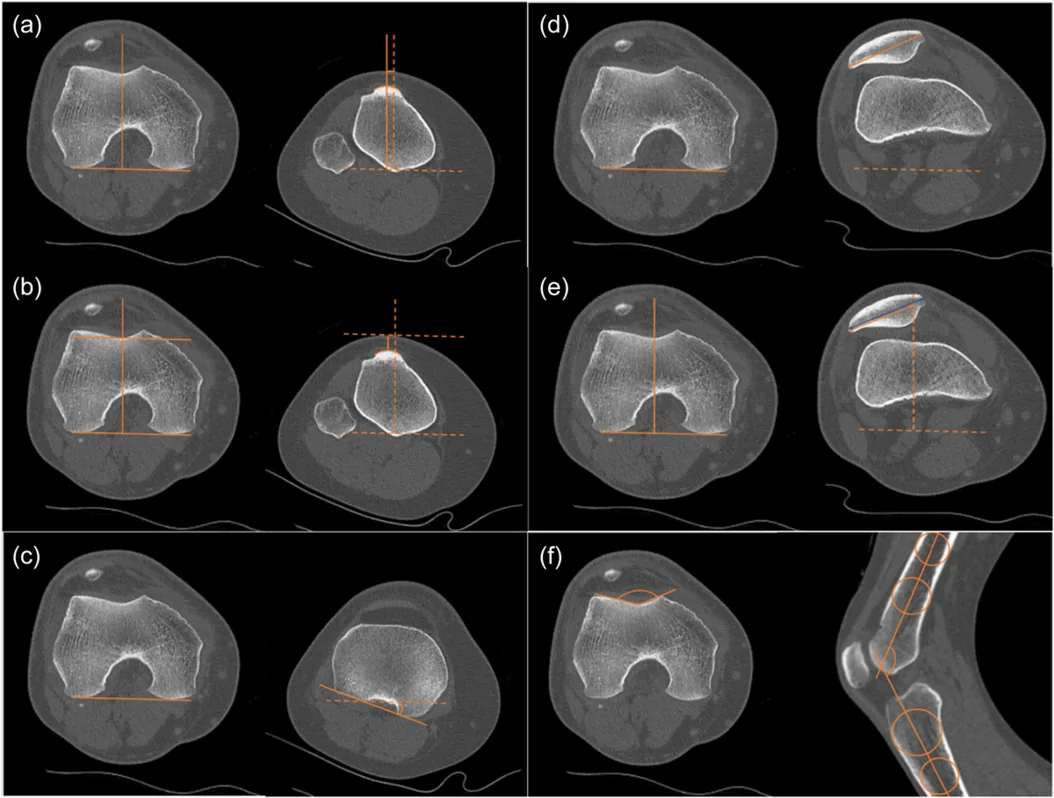
Illustration of anatomical measurements derived from dynamic CT imaging. (a) Anterior–posterior tibial tubercle–trochlear groove (apTTTG) distance, measured as the anteroposterior offset between the deepest point of the bony trochlear groove and the centre of the tibial tuberosity (orange arc) of the tibial tubercle, perpendicular to the posterior femoral condylar axis. (b) Axial tibial tubercle–trochlear groove (TT–TG) distance, defined as the mediolateral distance between the trochlear groove and centre of the tibial tuberosity (orange arc), both referenced to perpendicular lines from the posterior condylar axis. (c) Tibiofemoral rotation angle (TFRA) is calculated as the angle between the posterior femoral condylar line and the posterior tibial condylar line; positive values indicate external tibial rotation, and negative values indicate internal rotation. (d) Patellar tilt angle, measured between a line across the maximal transverse diameter of the patella and a line tangent to the posterior femoral condyles. (e) Patellar bisect offset, calculated as the percentage of the patellar width lateral to a midline perpendicular to the posterior condylar axis through the trochlear groove; greater values reflect increased lateralization. (f) Sulcus angle, defined as the angle between the medial and lateral trochlear facets at the deepest point of the trochlea (left), and knee flexion angle, measured on sagittal slices as the angle between the long axes of the femur and tibia (right). CT, computed tomography.

The apTTTG distance was defined as the anteroposterior offset between the deepest point of the trochlear groove, identified on the axial slice showing the Roman arch, and the most prominent point of the tibial tubercle, measured along a line perpendicular to the posterior femoral condylar axis (Figure 1a) [14, 26]. A positive apTTTG value indicated that the trochlea was located anterior to the tibial tubercle [12, 26, 28].

### TT–TG

The TT–TG distance was defined as the mediolateral offset between the deepest point of the trochlear groove, identified on the axial slice showing the Roman arch and the centre of the tibial tubercle, measured along lines perpendicular to the posterior femoral condylar axis (Figure 1b) [24].

### TFRA

TFRA was measured between the posterior condylar line of the femur and the posterior condylar line of the tibia (Figure 1c) [15]. A positive angle indicated external rotation of the tibia relative to the femur, while a negative angle reflected internal tibial rotation.

### Patella BO

The BO represents the percentage of the patella that is lateral to the centre of the trochlear groove. This was measured by drawing a reference line perpendicular to the posterior femoral condylar axis through the deepest point of the trochlear groove [5, 18]. The maximal transverse diameter of the patella was then overlaid, and the BO was calculated as the percentage of the patellar width positioned lateral to this midline reference (Figure 1e). Higher values indicated increased lateralization of the patella.

Patellar tilt was measured as the angle between a line along the maximal transverse diameter of the patella and a line tangent to the posterior femoral condyles (Figure 1d) [4, 10].

### Sulcus angle

The sulcus angle was defined on CT images as the angle between the medial and lateral trochlear facets, measured at the axial slice corresponding to the deepest point of the trochlear groove (Figure 1f), which was taken at the most superior axial level where the trochlear groove is fully covered by cartilage and the posterior femoral condylar cortices are visible [22].

### Knee flexion assessment

As an additional measurement, the knee flexion angle of the respective CT frame was measured on the mid‐sagittal image as the angle subtended by the long axes of the femur and tibia (Figure 1f).

### Statistical analysis

Continuous variables are reported as means ± standard deviations, and categorical variables as absolute and relative frequencies. To compare anatomical parameters across flexion states (extension, midflexion and flexion), the nonparametric Kruskal–Wallis test was used due to non‐normal distribution of values. Post hoc comparisons were performed using Dunn's test with Holm correction to adjust for multiple testing. Sex‐specific and side‐specific effects were assessed using mixed‐effects models including gender, side and flexion state.

To identify factors independently associated with the apTTTG distance, a multivariable linear mixed‐effects model was constructed including knee flexion angle, TFRA, patellar tilt, BO and sulcus angle as fixed effects. To account for the repeated measurements obtained from each subject, patient ID was included as a random intercept. Multicollinearity among fixed effects was assessed prior to model construction, and model assumptions were evaluated by inspection of residual plots. Model fit was assessed using likelihood‐based criteria (Akaike Information Criterion [AIC]) and statistical significance of individual fixed effects.

Inter‐rater reliability was assessed for all DKCT‐derived imaging measurements using two‐way random‐effects intraclass correlation coefficients (ICCs) for absolute agreement [ICC(2,1)] with 95% confidence intervals (CIs).

To further explore the relationship between knee flexion angle and apTTTG variation, an exploratory univariable linear regression analysis was performed. To assess potential nonlinear patterns, a quadratic regression model and a generalized additive model (GAM) using penalized regression splines were additionally fitted. Model performance was compared using the AIC and adjusted *R* ^2^, and the effective degrees of freedom (edf) of the smooth term in the GAM were reported to evaluate potential deviations from linearity.

To evaluate whether the study was adequately powered to detect the primary effect, a post hoc power analysis was performed based on the observed linear association between knee flexion angle and apTTTG (*R* = 0.75, *n* = 42 knees, *α* = 0.05). Based on this effect size, the achieved statistical power exceeded 0.99 for detecting a significant flexion‐dependent change in apTTTG, indicating that the sample size was sufficient for the primary endpoint.

A *p* value < 0.05 was considered statistically significant. All statistical analyses were performed using R (Version 4.2.3; R Foundation for Statistical Computing).

## RESULTS

A total of 42 knees from 21 patients were included in this study. The mean age was 22.5 ± 7.0 years (range, 15–40 years), and the mean body mass index (BMI) was 23.2 ± 4.5 kg/m^2^ (range, 16.7–36.1 kg/m^2^). The cohort consisted of 14 female and 7 male patients.

Changes in the key anatomical parameters are summarized in Table 1. Nonparametric testing using the Kruskal–Wallis test revealed significant differences across the three flexion positions (extension, midflexion and flexion) for all investigated parameters: apTTTG, flexion angle, sulcus angle, BO index, patellar tilt, TTTG and TFRA (all *p* < 0.001).

**Table 1:** Imaging measurements during different flexion grades.

	Extension	Midflexion	Flexion	
	Mean ± SD (range)	Mean ± SD (range)	Mean ± SD (range)	*p*
*All*				
Knee flexion (°)	3.62 ± 8.4 (−11 to 31.6)	27.7 ± 7.05 (15.7–48.6)	56.28 ± 9.1 (36.6–77.1)	**<0.0001**
apTTTG (mm)	−3.81 ± 5.71 (−13.8 to 6.4)	−8.82 ± 7.24 (−33.4 to 7.8)	−17.75 ± 8.36 (−30.2 to 17.9)	**<0.001**
TFRA (°)	−0.28 ± 6.06 (−14.8 to 9.1)	−7.87 ± 4.25 (−18.2 to 5.2)	−9.05 ± 4.15 (−18.1 to 1.2)	**<0.001**
TT–TG (mm)	14.82 ± 4.69 (3.3–23)	7.76 ± 3.72 (1–16.8)	6.69 ± 3.62 (1.3–16.4)	**<0.001**
Sulcus angle (°)	147.15 ± 10.94 (125.2–177.1)	135.35 ± 8.92 (120–160)	136.54 ± 8.43 (118–152)	**<0.001**
Patellar tilt (°)	17.84 ± 10.03 (0–40.1)	9.91 ± 5.03 (1–24.4)	8.75 ± 4.19 (0.5–18)	**<0.001**
Patella bisect offset (%)	79.02 ± 15.11 (53–131)	60.02 ± 7.77 (46–82)	59.55 ± 7.55 (45–86)	**<0.001**
*Symptomatic*				
Knee flexion (°)	3.82 ± 8.4 (−11 to 31.6)	28.11 ± 6.83 (17–47.2)	56.78 ± 11.41 (36.6–77.1)	**<0.0001**
apTTTG (mm)	−3.80 ± 5.82 (−13.8 to 6.2)	−7.93 ± 7.09 (−26.7 to 7.8)	−16.93 ± 10.2 (−30.2 to 17.9)	**<0.001**
TFRA (°)	−0.28 ± 6.06 (−14.8 to 9.1)	−7.52 ± 5.21 (−17 to 5.2)	−8.74 ± 4.46 (−18.1 to 1.2)	**<0.001**
TT–TG (mm)	16.14 ± 4.42 (5.1–23)	8.57 ± 3.67 (1–16)	7.2 ± 3.61 (1.7–14.1)	**<0.001**
Sulcus angle (°)	147.95 ± 9.13 (129.7–163.6)	135.67 ± 9.2 (120–151)	138.45 ± 6.08 (127–152)	**<0.001**
Patellar tilt (°)	20.4 ± 11.2 (2.2–40.1)	10.65 ± 5.91 (1–24.4)	9.08 ± 4.65 (0.5–17.6)	**<0.001**
Patella bisect offset (%)	85.38 ± 19.1 (54–131)	62 ± 9.55 (46–82)	60.71 ± 8.32 (45–86)	**<0.001**
*Contralateral*				
Knee flexion (°)	3.42 ± 7.13 (−9.2 to 19.6)	27.3 ± 7.26 (15.7–48.6)	55.79 ± 11 (38.2–74)	**<0.0001**
apTTTG (mm)	−3.82 ± 5.59 (−13.7 to 6.4)	−9.7 ± 7.4 (−33.5 to 7.2)	−18.57 ± 6.5 (−29.9 to 6.7)	**<0.001**
TFRA (°)	−1.82 ± 5.99 (−14.8 to 6.8)	−8.22 ± 4.96 (−18.2 to 0)	−9.37 ± 3.83 (−1.8 to 1.9)	**<0.001**
TT–TG (mm)	13.5 ± 4.95 (3.3–20.6)	6.95 ± 3.78 (1.2–16.8)	6.17 ± 3.63 (1.3–16.4)	**<0.001**
Sulcus angle (°)	146.34 ± 12.76 (125.2–177.1)	135.02 ± 8.64 (120–160)	134.62 ± 6.54 (118–145)	**<0.001**
Patellar tilt (°)	15.28 ± 8.49 (0–36.5)	9.17 ± 4.15 (1.3–16.9)	8.43 ± 4.23 (1.6–18)	**<0.001**
Patella bisect offset (%)	72.67 ± 11.11 (53–100)	58.05 ± 5.99 (47–71)	58.38 ± 6.79 (49–72)	**<0.001**

*Note*: Data are presented as mean ± SD (range). Bold *p* value indicates statistical significance (*p* < 0.05).

Abbreviations: apTTTG, anterior–posterior tibial tubercle–trochlear groove distance; SD, standard deviation; TFRA, tibiofemoral rotation angle; TT–TG, tibial tubercle–trochlear groove distance.

Post hoc analysis using Dunn's test with Holm correction revealed distinct patterns across the assessed parameters. For apTTTG and knee flexion angle, significant differences were observed between all three flexion states (adjusted *p* values < 0.01).

BO also differed significantly between all flexion states (adjusted *p* < 0.01). Patellar tilt showed significant reductions from extension to both midflexion and flexion (*p* < 0.001), but no significant difference between midflexion and flexion (*p* = 0.39).

Sulcus angle demonstrated significant differences between extension and both midflexion and flexion (*p* < 0.001), without a significant difference between midflexion and flexion (*p* = 0.52). Similarly, TT–TG and TFRA significantly decreased from extension to midflexion and flexion (both *p* < 0.001), but showed no additional significant differences between midflexion and flexion (TT–TG: *p* = 0.25; TFRA: *p* = 0.33).

Inter‐rater reliability was good to excellent for all measured parameters. The ICC for apTTTG was 0.94 (95% CI, 0.91–0.96). Excellent reliability was also observed for TT–TG distance (ICC, 0.93; 95% CI, 0.90–0.95), knee flexion angle (ICC, 0.96; 95% CI, 0.94–0.97), patellar tilt (ICC, 0.91; 95% CI, 0.88–0.94) and sulcus angle (ICC, 0.90; 95% CI, 0.86–0.93). Reliability was good to excellent for TFRA (ICC, 0.89; 95% CI, 0.85–0.92) and BO (ICC, 0.88; 95% CI, 0.84–0.91).

Side‐specific analyses were performed as secondary exploratory analyses. In full extension, symptomatic knees demonstrated significantly higher BO (85.4 ± 19.6% vs. 72.7 ± 11.4%, *p* < 0.001), greater patellar tilt (20.4 ± 11.5° vs. 15.3 ± 9.1°, *p* = 0.0045), higher TT–TG distance (16.1 ± 4.7 vs. 14.0 ± 5.0 mm, *p* = 0.041) and increased external tibiofemoral rotation (1.26 ± 6.27° vs. −1.82 ± 6.14°, *p* = 0.02) compared with contralateral knees, whereas no significant side differences were observed for apTTTG (−3.74 ± 5.97 vs. −3.88 ± 5.78 mm, *p* = 0.94), flexion angle (3.82 ± 8.61° vs. 3.42 ± 7.31°, *p* = 0.85) or sulcus angle (148 ± 9.35° vs. 146 ± 13.1°, *p* = 0.47). With increasing knee flexion, side differences progressively diminished across all parameters. Correspondingly, no significant side‐by‐flexion interaction was identified for apTTTG (*p* = 0.809), flexion angle (*p* = 0.977), sulcus angle (*p* = 0.563), patellar tilt (*p* = 0.152), TT–TG (*p* = 0.713) or tibiofemoral rotation (*p* = 0.305), indicating parallel flexion‐dependent behaviour between symptomatic and contralateral knees; BO was the only parameter showing a significant side‐by‐flexion interaction (*p* = 0.026), reflecting a side‐specific dynamic alteration that was most pronounced in extension and converged at higher flexion angles.

Sex‐specific effects were evaluated using mixed‐effects models including sex, side and flexion state. No significant sex × side × flexion interaction was observed for any parameter (all *p* ≥ 0.306), indicating comparable flexion‐dependent side differences between males and females. For apTTTG, males showed descriptively more negative values than females; however, the significant unadjusted sex main effect (*χ* ^2 ^= 5.16, *p* = 0.023) was no longer significant after adjustment for body height (*χ* ^2 ^= 2.04, *p* = 0.154). In contrast, TT–TG showed a significant sex main effect (*χ* ^2 ^= 11.2, *p* < 0.001), which remained significant after height adjustment, although attenuated (*χ* ^2 ^= 4.56, *p* = 0.033). All other measurements showed no significant sex main effects and remained non‐significant after adjustment for body height.

To evaluate factors independently associated with apTTTG, a multivariable linear mixed‐effects model was constructed including knee flexion angle, TFRA, patellar tilt, BO and sulcus angle as fixed effects. To account for repeated measurements within patients, subject ID was included as a random intercept.

After adjustment for all covariates, knee flexion angle remained the only significant independent predictor of apTTTG (*β* = –0.28 mm/°, 95% CI –0.34 to –0.22; *p* < 0.001), indicating that each degree of knee flexion was associated with a reduction of approximately 0.28 mm in apTTTG. None of the other variables demonstrated an independent association with apTTTG, including TFRA (*p* = 0.88), patellar tilt (*p* = 0.20), BO (*p* = 0.50) and sulcus angle (*p* = 0.99).

**Figure 2:**
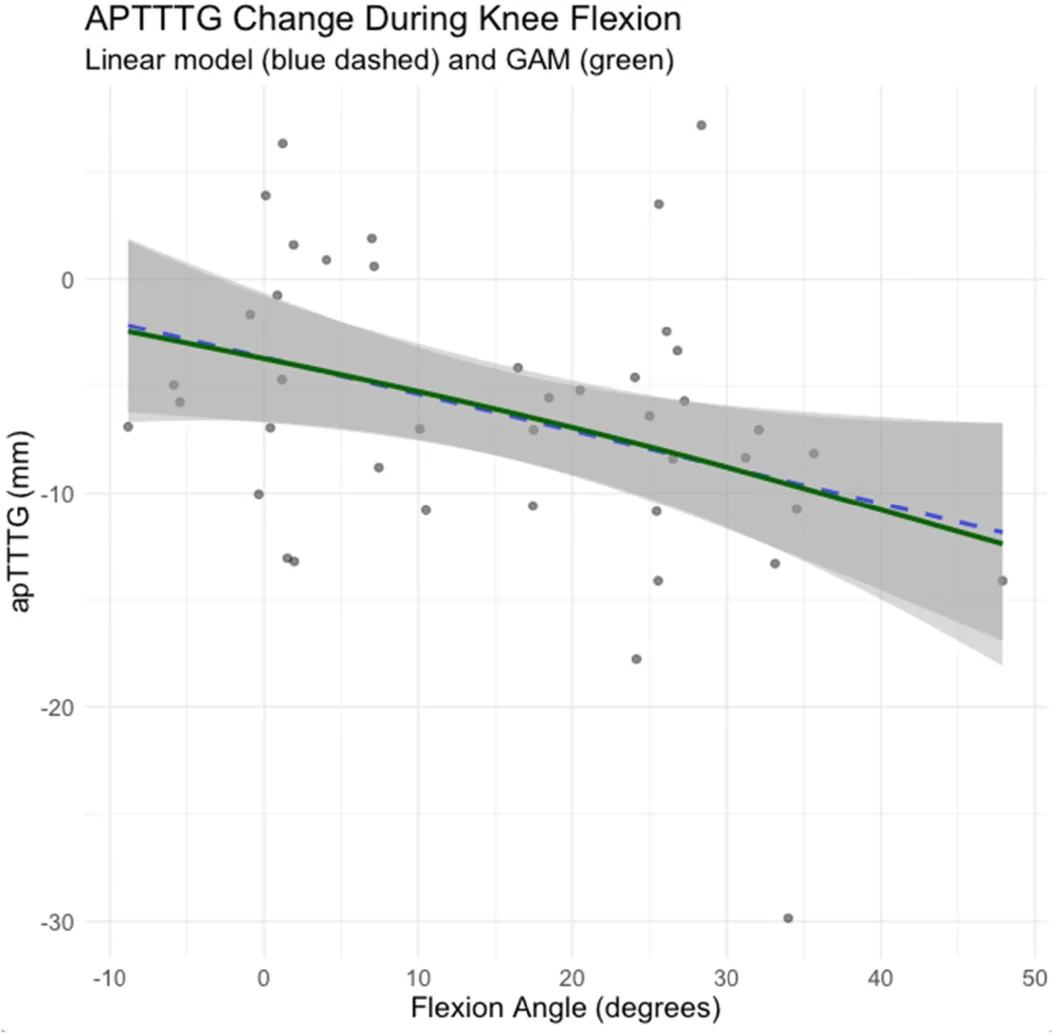
Dynamic changes in apTTTG during knee flexion, plotted against flexion angle. Each subplot compares a linear regression model (blue dashed line) with a generalized additive model (GAM, green solid line), including 95% confidence intervals (grey shaded areas). apTTTG, anterior‐posterior tibial tubercle–trochlear groove distance.

To further assess the nature of this relationship, an exploratory linear regression was performed using knee flexion angle as the sole predictor, revealing a significant inverse association with apTTTG (*β* = −0.17, *p* = 0.02), accounting for 13% of the variance. Neither a quadratic model (*p* = 0.24) nor a GAM provided a substantially improved fit. Although the GAM yielded a statistically significant smooth term (*p* = 0.03), the edf (edf = 1.13) indicated a near‐linear relationship (Figure 2).

## DISCUSSION

The most important finding of this study was that apTTTG decreased progressively with increasing knee flexion and demonstrated a near‐linear relationship across the investigated flexion range. Knee flexion angle was the only independent predictor of apTTTG in the multivariable model, whereas TFRA, patellar tilt, BO and sulcus angle were not independently associated with apTTTG. In contrast, several established patellofemoral alignment parameters, including TT–TG, TFRA, patellar tilt, BO and sulcus angle, showed their most pronounced changes between extension and midflexion, with less change thereafter. Side‐specific analyses did not demonstrate a significant side‐by‐flexion interaction for apTTTG, indicating a comparable flexion‐dependent pattern between symptomatic and contralateral knees. These findings suggest that apTTTG is a position‐dependent sagittal‐plane measurement and that knee flexion angle should be considered when interpreting apTTTG in patients evaluated for PFI.

The flexion‐dependent behaviour of apTTTG indicates that this parameter should not be interpreted as a fixed anatomic constant. Rather, apTTTG reflects the dynamic sagittal relationship between the tibial tubercle and the trochlear groove. In the present study, apTTTG became progressively more negative from extension to flexion, indicating a relative anterior shift of the tibial tubercle in relation to the trochlear reference point during knee flexion. This finding is clinically relevant because differences in knee flexion during CT or MRI acquisition may influence absolute apTTTG values and may reduce comparability between studies, imaging modalities or preoperative and postoperative examinations.

The present findings also help to clarify the currently inconsistent literature regarding the flexion dependency of apTTTG/sTTTG. Ackermann et al. found no significant association between knee flexion angle and sTTTG on MRI or CT in patients with patellar instability [2]. However, their analysis was based on standard static imaging and a limited range of knee flexion, which may have reduced the ability to detect flexion‐dependent changes, particularly once the patella was already engaged within the trochlea. In contrast, the present study used DKCT to assess different functional stages of patellofemoral engagement and demonstrated a progressive, near‐linear change in apTTTG with increasing flexion. Thus, the findings support the concept that dynamic assessment may reveal position‐dependent behaviour that is not fully captured by conventional static imaging.

Another important finding is that apTTTG appears to capture a distinct aspect of patellofemoral geometry, independent of other established alignment indices. In the present analysis, apTTTG was not independently associated with conventional axial TT–TG distance, trochlear morphology as assessed by sulcus angle or patellar position as assessed by patellar tilt and BO. This is consistent with previous work showing that patients with patellofemoral cartilage lesions demonstrated different apTTTG values compared with controls, despite no significant differences in axial TT–TG, trochlear groove angle or patellar height [12]. Lansdown et al. similarly reported a more posteriorly positioned tibial tubercle, reflected by a larger posterior sTT–TG offset, in patients with patellofemoral chondral lesions [14]. These findings suggest that sagittal tibial tubercle position may represent an independent alignment factor that is not fully captured by conventional axial or rotational parameters.

The dynamic behaviour of apTTTG differed from several other patellofemoral alignment parameters. TT–TG, TFRA, patellar tilt and BO showed their largest changes between extension and midflexion, likely reflecting the transition from a relatively disengaged patella in extension to increasing trochlear engagement during early flexion. In contrast, apTTTG continued to change across the assessed flexion arc and demonstrated a near‐linear relationship with knee flexion angle. This proportional behaviour may be particularly relevant for imaging interpretation, as even relatively small differences in knee flexion during acquisition may influence absolute apTTTG values [26, 32].

The apparent flexion‐dependent change in sulcus angle should not be interpreted as a true change in trochlear morphology. Rather, it likely reflects the three‐dimensional anatomy of the trochlea and dynamic slice selection during DKCT. With increasing flexion, the patella engages more distal and deeper portions of the trochlea, whereas extension may sample a more proximal and flatter region. This may explain why the measured sulcus angle appeared to decrease with flexion.

From a clinical imaging perspective, the main implication of this study is that knee flexion angle matters when interpreting apTTTG in the setting of PFI. Although apTTTG has been discussed as a potentially relevant parameter for sagittal patellofemoral alignment, patellofemoral contact mechanics and surgical planning, the present study did not evaluate the effect of TTO or any other surgical intervention. Therefore, the current data should not be interpreted as evidence supporting a specific operative correction. Instead, they emphasize the need for standardized knee positioning and careful documentation of knee flexion angle when apTTTG is measured. This is particularly important when apTTTG values are compared between different studies, imaging protocols or longitudinal examinations [2].

The objective of this study was to evaluate the dynamic behaviour of apTTTG in knees from patients evaluated for PFI, rather than to compare distinct clinical subgroups. The cohort therefore included both untreated unilateral patellar instability and patients with failed soft‐tissue stabilization. Although these entities differ in clinical history, both represent knees with ongoing instability and uncorrected osseous morphology, allowing assessment of patellofemoral alignment under clinically relevant pathological conditions.

The contralateral limb was included as an internal reference and not as a healthy control group. PFI frequently demonstrates bilateral anatomical risk factors; therefore, contralateral knees in this cohort were not assumed to be anatomically normal. Instead, they represented morphologically at‐risk but clinically stable knees within the same individuals. This approach reduces interindividual variability related to sex, body habitus and osseous morphology. Importantly, the primary analysis focused on flexion‐dependent changes within knees, and side‐specific comparisons were considered secondary exploratory analyses. The absence of a significant side‐by‐flexion interaction for apTTTG supports the interpretation that the observed flexion‐dependent behaviour was not driven by clinical side status.

Several limitations must be acknowledged. First, this investigation evaluated three flexion positions: extension, midflexion and flexion. Therefore, the behaviour of apTTTG beyond approximately 60° remains uncertain. It is possible that additional changes occur at deeper flexion angles; however, these changes may be less clinically relevant because the patella is typically more fully engaged within the trochlea at higher degrees of flexion. Second, the findings cannot be generalized to skeletally immature patients, as only individuals with closed physes were included. Third, the results are derived from a specific patient population and imaging protocol; therefore, variations in acquisition technique, patient positioning or imaging modality may influence absolute apTTTG values, even if the overall flexion‐dependent trend remains similar [2].

Additional methodological limitations should also be considered. No formal a priori sample size calculation was performed before study initiation. Although the observed primary effect was large and the post hoc power analysis indicated adequate power for the primary outcome, the absence of an a priori power calculation limits methodological transparency. Furthermore, the number of included patients was modest. Although bilateral DKCT measurements in 21 individuals resulted in 42 knees and repeated observations across flexion states, this sample size may not fully capture the variability of patellofemoral alignment across a broader population. Finally, although patients with clinical or MRI‐based evidence of ACL or PCL insufficiency were excluded, posterior tibial slope and posterior femoral condyle offset were not included in the present analysis. Because these parameters may influence sagittal tibiofemoral translation, future studies should evaluate their contribution to apTTTG behaviour during knee flexion.

Future studies should further investigate the clinical relevance of apTTTG by correlating dynamic measurements with symptoms, patient‐reported outcomes, cartilage status and surgical results. Prospective longitudinal studies incorporating standardized flexion‐angle documentation may help determine whether apTTTG thresholds should be interpreted relative to knee position. In addition, future work should evaluate whether changes in apTTTG after surgical correction are associated with functional improvement, symptom relief or long‐term cartilage preservation.

## CONCLUSION

The apTTTG distance decreases linearly with increasing knee flexion, whereas other patellofemoral alignment parameters change predominantly between extension and midflexion.

## AUTHOR CONTRIBUTIONS

Sebastian Schmidt, Alexander Bumberger and Miho J. Tanaka provided the concept of this study. Sebastian Schmidt and Alexander Bumberger were responsible for data collection, provided ongoing results, figures and tables and wrote the manuscript. Alexander Bumberger and Miho J. Tanaka revised the manuscript. The authors read and approved the final manuscript.

## FUNDING INFORMATION

The authors have no funding to report.

## CONFLICT OF INTEREST STATEMENT

The authors declare no conflict of interest.

## ETHICS STATEMENT

This study was conducted in accordance with the principles of the Declaration of Helsinki and approved by the Institutional Review Board of Mass General Brigham (Protocol Number 2016P001295).

## Data Availability

The data that support the findings of this study are not openly available due to reasons of sensitivity but are available from the corresponding author upon reasonable request.
